# Spontaneous Coronary Artery Dissection: Pitfalls of Angiographic Diagnosis and an Approach to Ambiguous Cases

**DOI:** 10.1016/j.jcin.2021.06.027

**Published:** 2021-08-23

**Authors:** David Adlam, Marysia S. Tweet, Rajiv Gulati, Deevia Kotecha, Praveen Rao, Alistair J. Moss, Sharonne N. Hayes

**Affiliations:** a Department of Cardiovascular Sciences and NIHR Leicester Biomedical Research Centre, University of Leicester, Leicester, United Kingdom; b Department of Cardiovascular Medicine, Mayo Clinic, Rochester, Minnesota, USA

**Keywords:** angiography, computed tomography, differential diagnosis, intravascular ultrasound, optical coherence tomography, spontaneous coronary artery dissection, women, ACS, acute coronary syndrome(s), CTCA, computed tomographic coronary angiography, FMD, fibromuscular dysplasia, IVUS, intravascular ultrasound, LAD, left anterior descending coronary artery, OCT, optical coherence tomography, PCI, percutaneous coronary intervention, SCAD, spontaneous coronary artery dissection

## Abstract

Spontaneous coronary artery dissection (SCAD) is a pathophysiologically distinct cause of acute coronary syndromes (ACS). It is increasingly recognized that optimal management is different from that for atherosclerotic ACS and that a SCAD diagnosis has specific long-term prognostic and therapeutic implications. Accurate diagnosis is therefore essential to ensure the best treatment of patients. At present this relies on the recognition of typical features of SCAD identified on invasive coronary angiography. Although most SCAD can be readily distinguished angiographically from alternative causes of ACS, false positive and false negative diagnoses remain common. In particular, sometimes non-SCAD presentations, including atherothrombosis, takotsubo cardiomyopathy, coronary embolism, coronary vasospasm, contrast streaming, and myocardial infarction with nonobstructive coronary arteries, can mimic angiographic features usually associated with SCAD. The authors present the combined experience from European and US SCAD referral centers reviewing the classical angiographic appearances of SCAD, presenting potential diagnostic pitfalls and exemplars of SCAD mimickers. The authors further review the benefits and limitations of intracoronary imaging in the context of SCAD. Finally, the authors discuss the investigation of ambiguous cases and an approach to minimize misdiagnosis in difficult cases.

## Highlights

• Accurate diagnosis of SCAD is critical as recommended interventional management differs from atherosclerotic ACS. For SCAD, a conservative approach is favored over percutaneous coronary intervention where possible.
• SCAD diagnosis is based on invasive angiography. While the existing angiographic classification helps with recognition of common presentations, some SCAD appearances are not easily classified and sometimes other non-SCAD diagnoses can generate angiographic SCAD mimickers.
• The authors present the potential pitfalls in the angiographic diagnosis of SCAD to help clinicians recognize areas of diagnostic uncertainty. They propose an approach centered around the careful assessment of pre- and post-angiographic probability coupled with the judicious use of intracoronary imaging and other follow-up investigations to maximize diagnostic accuracy.

Spontaneous coronary artery dissection (SCAD) is recognized as an important cause of acute coronary syndrome (ACS) leading to myocardial infarction (1, 2, 3, 4). It is caused by hematoma formation within the tunica media of the coronary vessel wall, leading to the development of a false lumen that tracks both longitudinally and circumferentially. There is increasing evidence that in most cases, this hematoma arises de novo within the vessel wall, rather than as a consequence of a primary endothelial-intimal tear or flap (3,5,6). Compression of the true lumen leads to coronary insufficiency, myocardial infarction, and in some cases ventricular arrhythmia (7). Accurate diagnosis is critical, as management of SCAD differs compared with that for ACS of atherosclerotic etiology both in the cardiac catheterization laboratory and afterward. For example, percutaneous coronary intervention (PCI) in SCAD is associated with high rates of complications and lower rates of angiographic success, whereas conservative management is associated with complete coronary healing in most cases (7, 8, 9). For this reason, both European and American consensus documents recommend a conservative strategy when practicable (1,2). The diagnosis of SCAD can be challenging. At present, there is no biomarker that reliably differentiates SCAD from atherosclerosis. Noninvasive diagnosis by computed tomographic coronary angiography (CTCA) is not routinely recommended, because of its lower spatial resolution, which limits assessment of the more distal coronary territories that are frequently affected by SCAD (10). As such, the diagnosis continues to rely upon recognition of characteristic features on invasive angiography. The aim of this review is to revisit the diagnostic features of SCAD, present potential pitfalls including SCAD mimickers, and suggest an approach to optimize accurate diagnosis.

## Pretest Probability: Before Angiography

Before the patient arrives in the cardiac catheterization laboratory, there are a number of characteristics that influence the pretest probability of SCAD. For example, almost all patients with SCAD present with ACS. Biomarkers of myocardial injury (especially serial monitoring of high-sensitivity troponin [11]) are almost invariably elevated, except perhaps in cases in which presentation is very early or has been delayed. A nonacute presentation should therefore raise the level of diagnostic doubt.

Patients with SCAD are overwhelmingly female, with male SCAD occurring in about 10% of cases in most series (9,12,13). SCAD has been reported as the cause of up to 35% of ACS events in women younger than 50 years (14, 15, 16) and 23% to 68% of pregnancy-associated ACS (17,18). The relative rarity of men diagnosed with SCAD compared with those with atherosclerosis warrants a higher index of suspicion of potential SCAD in men. Cases of SCAD in older patients are increasingly recognized (3), with a mean age in a recent prospective series of 52 years (12). However, SCAD is uncommon in very young adults (<25 years of age), especially outside the context of pregnancy or hereditary connective tissue disorders, and is also uncommon in very old patients (>80 years of age). Presentations falling outside this age range should therefore be more carefully scrutinized before a diagnosis of SCAD is confirmed. It is worth noting that up to 90% of SCAD cases reportedly occur in women between 47 and 53 years of age (4). The presence or absence of atherosclerotic risk factors is not very useful as a guide to the likelihood of SCAD. It is important to appreciate that although risks are lower than in atherosclerotic patients, patients with SCAD are not “free” of risk factors, as is sometimes reported. Hypertension occurs in about 30% of patients with SCAD, although established diabetes is rare (12). Conversely, because atherosclerosis is at least an order of magnitude more common than SCAD, it remains the most probable cause of ACS, even in patients with few risk factors (3).

SCAD is associated with a small number of known genetic disorders (19,20). A recent gene sequencing study showed that 3.5% of patients with SCAD had causal or likely pathogenic rare genetic variants, mostly in genes associated with other known disorders (eg, vascular Ehlers-Danlos, Loeys-Dietz, or adult polycystic kidney disease) (21). Patients presenting with ACS who are known to have these disorders or with family histories or suggestive clinical features should trigger consideration of a potential SCAD diagnosis.

Symptoms at the time of SCAD presentation are similar to those occurring with other causes of ACS and therefore are not a useful diagnostic discriminator (22). In some patients, potential trigger exposures have been identified, such as emotional or physical stressors (23). Where clear-cut, such as the onset of symptoms during isometric exercise, this may increase the probability of a SCAD diagnosis. However, trigger exposures may also occur in other causes of ACS, such as emotional stress with takotsubo syndrome (24) or exercise and atherosclerotic plaque rupture (25).

Although clearly patient factors cannot by themselves confirm or refute a diagnosis of SCAD, these factors can help clinicians titrate their levels of suspicion for SCAD as the underlying diagnosis.

## Angiographic Diagnosis of SCAD

**Figure 1:**
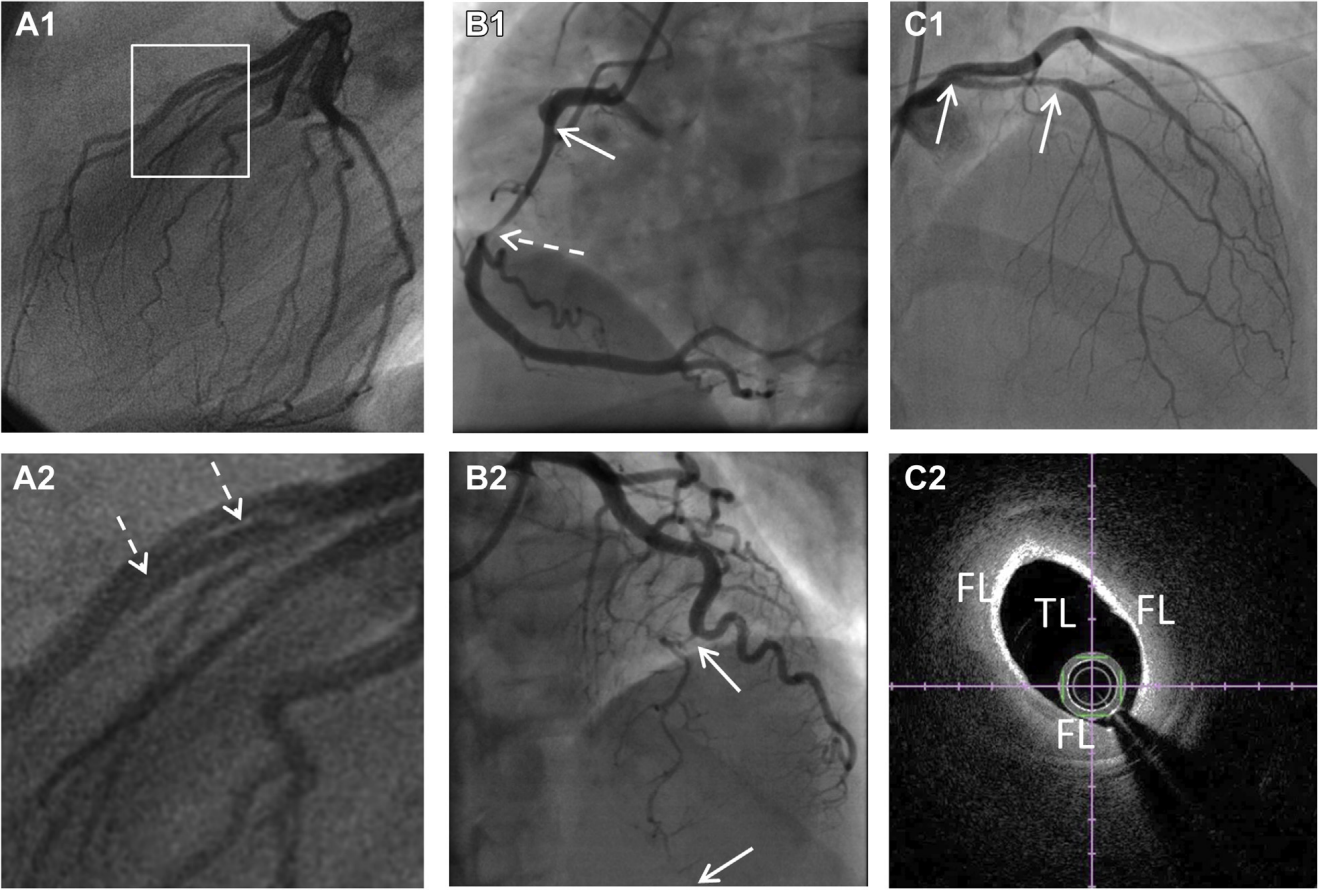
Classical Appearances of SCAD as Defined by the Yip-Saw Classification (27) Type 1 spontaneous coronary artery dissection (SCAD) **(A1)** showing dual-lumen appearance (magnified with **dotted arrows** in **A2**). Type 2a SCAD **(B1)** with long smooth narrowing **(arrows)** tapering distally **(dotted arrow)** before recrudescence of a normal artery. Type 2b SCAD **(B2)** with long narrowing extending distally. Type 3 SCAD mimics an atherosclerotic stenosis **(arrows) (C1)** and can be distinguished only by intracoronary imaging **(C2)**. FL = false lumen; TL = true lumen.

Invasive coronary angiography remains the most important diagnostic modality in suspected SCAD and allows an accurate diagnosis in the majority of patients. SCAD occurs most commonly in the left anterior descending coronary artery (LAD) and in mid to distal coronary segments (1, 2, 3, 4). Angiographic classifications or descriptions have been developed to aid in pattern recognition of typical angiographic appearances of SCAD (26,27). The Yip-Saw classification was developed to aid in diagnostic pattern recognition of SCAD and divides angiographic features into 3 types (Figure 1) (27). Identifying type 1 SCAD (Figures 1A1 and 1A2), where contrast penetrates into the false lumen(s), giving a dual-lumen appearance sometimes with localized extraluminal dye “hang-up” following contrast clearance, is important, as it is pathophysiologically distinct from types 2 and 3. Type 1 appearances account for fewer than one-third of angiographic presentations and likely develop later in the disease course (probably as a result of decompression of the false lumen hematoma into the true lumen) (6). Type 1 SCAD is associated with a lower risk for clinical progression (if managed conservatively) and of PCI complications (if managed with revascularization) (6,28). Types 2 and 3 SCAD describe different appearances of intramural hematoma. Type 2 SCAD is the most common appearance (12), characterized by a long smooth stenosis. In type 2a, there is restoration of a normal vessel distal to the dissection, often with the tightest stenosis at the distal extent of the false lumen (Figure 1B1). In type 2b SCAD, the narrowing continues into the most distal angiographically visible segments (Figure 1B2). Type 3 SCAD is described as a lesion that mimics the appearances of focal atherosclerotic disease and cannot be definitively determined as SCAD on angiographic images without recourse to intracoronary imaging (Figures 1C1 and 1C2). In these cases, careful consideration of the pretest probability of SCAD (as described earlier) supported by other suggestive angiographic features, such as increased coronary tortuosity (29) or minimal nonculprit atherosclerotic plaque, can help identify cases in which further imaging is required to exclude SCAD.

**Figure 2:**
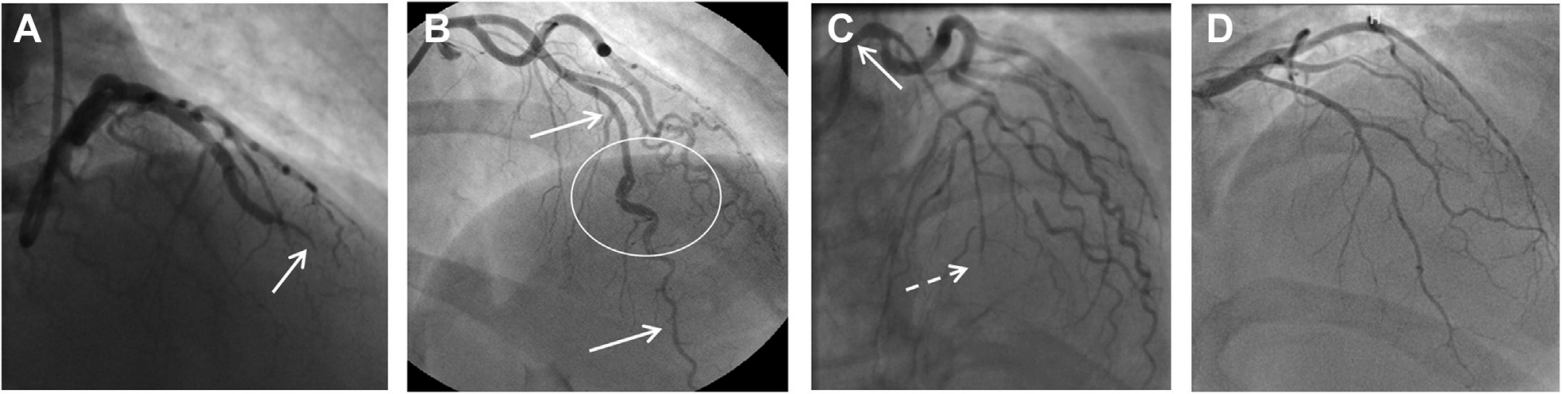
Spontaneous Coronary Artery Dissection Appearances Falling Outside the Yip-Saw Classification **(A)** Vessel occlusion (type 4 spontaneous coronary artery dissection) with upstream tapering **(arrow)** and otherwise normal coronary arteries. **(B)** Hybrid appearance with a segment of dual-lumen appearance **(circled)** within a long segment of stenosis from intramural hematoma **(arrows)**. **(C)** Extensive dissection extending from left mainstem **(arrow)** into the left anterior descending coronary artery and its branches, culminating in vessel occlusion **(dotted arrow)**. **(D)** Appearance of general vessel narrowing confirmed on optical coherence tomography as extensive intramural hematoma.

The Yip-Saw classification has aided in the angiographic recognition of type 2 SCAD in particular. Other approaches to aid in pattern recognition of SCAD angiograms have also been described (Supplemental Figure 1) (26,30,31). However, like any attempt at classification, these approaches are focused primarily on the most common angiographic presentations and therefore have limitations. For example, SCAD leading to vessel occlusions does not fit easily into these classifications. For these reasons, a modification has been proposed to add SCAD type 4, which is defined as vessel occlusions that do not meet the criteria for types 1 to 3 (Figure 2A) (32). Additionally, these classifications work less well to describe very extensive proximal dissections (Figure 2B), those with hybrid appearances (Figure 2C), and those with diffuse nonfocal narrowing (Figure 2D). It is important to recognize that the full spectrum of angiographic presentations in SCAD includes these less classical appearances.

Recent research has demonstrated that the common genetic risk variants associated with SCAD provide some protection from atherosclerotic disease (33, 34, 35). Extensive atheroma on angiography is correspondingly rare, although nonobstructive plaque may occur (36). Other coronary abnormalities, including increased tortuosity (29), have been described in patients with SCAD and may represent associated coronary arteriopathies (37), although an angiographic string of beads akin to the appearance of extracoronary fibromuscular dysplasia (FMD) appears rare (36). Intramural hematoma in patients with SCAD is frequently bounded at its proximal and distal extent by branch points, which seem to provide some resistance to further axial extension (26,30). This is distinct from atheroma, which has a predilection for bifurcation points. One additional key feature that can help in the differential diagnosis of SCAD is the degree of luminal thrombus. SCAD arises from external compression of the true lumen, and luminal thrombus is a less common feature when this has been assessed angiographically (9) or with optical coherence tomography (OCT) (5). Although thrombus can occur in occlusive SCAD or in association with fenestrations connecting true and false lumens, the presence of substantial luminal thrombus or evidence of downstream embolization of thrombus should lead to reconsideration of alternative diagnoses.

## Differential Diagnosis and Ambiguous Cases

Although angiographic features in SCAD are frequently characteristic, there are a number of potential pitfalls, important differential diagnoses and angiographic mimics to consider.

### Normal with streaming or vasospasm

**Figure 3:**
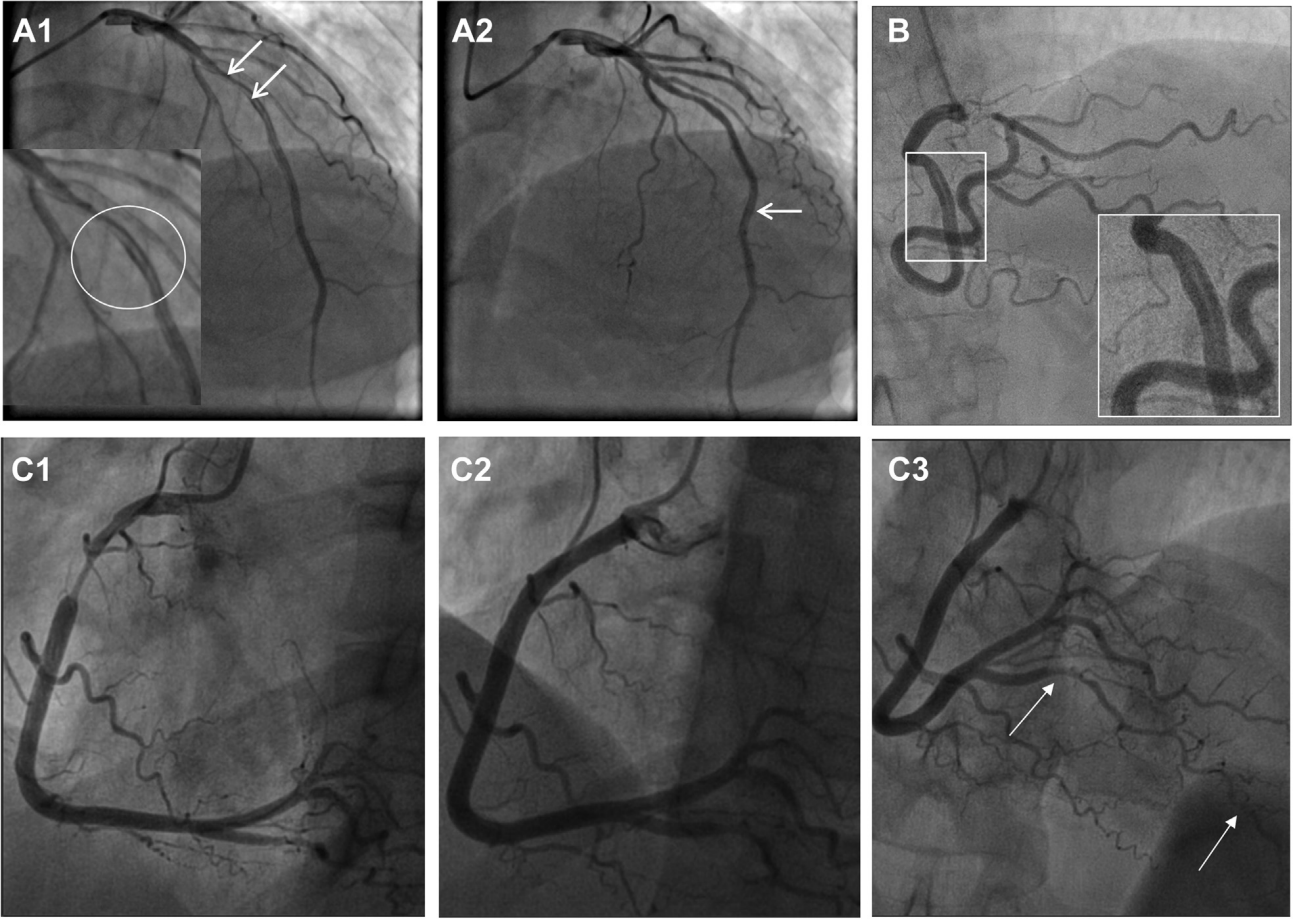
Normal Coronary SCAD Mimics **(A1)** Contrast streaming in the mid left anterior descending coronary artery (LAD) after a short stenosis gives the appearance of type 1 spontaneous coronary artery dissection (SCAD) **(circled and arrows)**. **(A2)** A few frames later, as more contrast is injected, this appearance is lost upstream, but an apparent dual-lumen appearance now appears more distally **(arrow)**. **(B)** A mid right coronary artery (RCA) probable type 1 SCAD is shown for comparison; note that the lack of heterogeneity in the dark contrast-opacified vessel makes streaming unlikely. This patient also had confirmed LAD SCAD. **(C1)** Catheter-induced coronary spasm of the RCA is relieved by intracoronary nitrate **(C2)**. Subsequent assessment of the distal vessel confirms a type 2b dissection of the posterior descending branch **(arrows, C3)**.

Contrast flow patterns can sometimes give the appearance of a linear filling defect (Figures 3A1 and 3A2), although it is usually straightforward to distinguish this from type 1 SCAD (Figure 3B) with a more fulsome coronary injection. Coronary artery vasospasm can generate long smooth stenoses, mimicking type 2 SCAD (Figures 3C1 to 3C3). Administration of intracoronary nitrate, when blood pressure permits, will usually relieve spasm (Figure 3C2), but caution is required, as there is often an element of vasospasm associated with SCAD.

### Atherosclerosis

**Figure 4:**
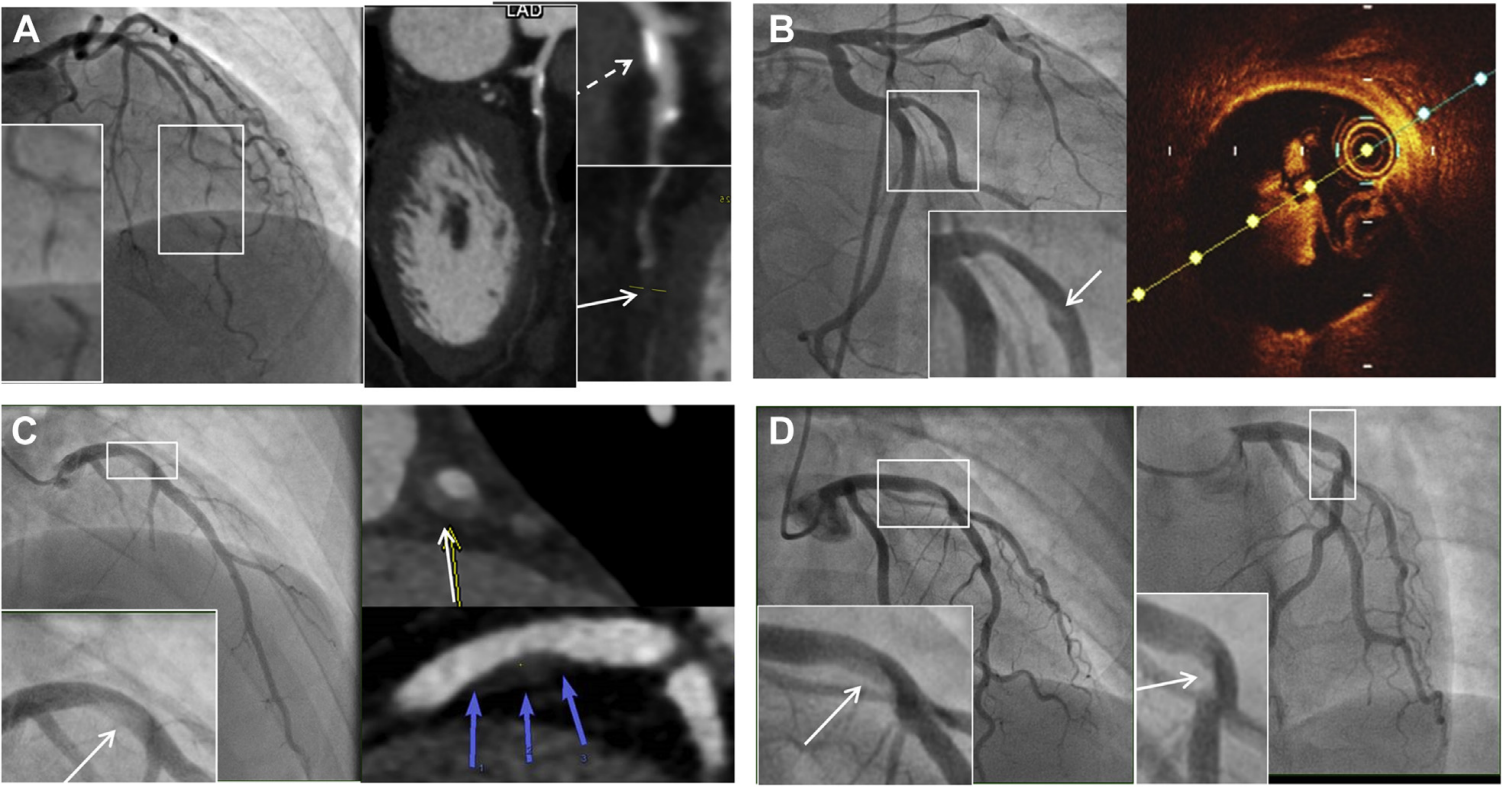
Atherosclerosis Versus Spontaneous Coronary Artery Dissection **(A)** A patient with angiographic appearances in keeping with Type 2a SCAD **(box)** but who continued to experience typical exertional angina at follow-up triggering reassessment by CTCA. This confirmed mixed calcific atherosclerotic plaque **(dotted arrow)** with persisting stenosis **(arrow). (B)** A stenosis with downstream filling defect **(box and arrow)** in the obtuse marginal branch of the circumflex. OCT imaging suggests this appearance is due to a tongue of organized thrombus arising from an area of plaque erosion extending within the downstream lumen. **(C)** A young man with no cardiovascular risk factors who developed chest pain during sport. Angiography showed a hazy linear abnormality in the mid left anterior descending coronary artery (LAD) **(box)**. Computed tomographic angiography demonstrated positively remodeled lipid-rich atherosclerotic plaque. A similar angiographic ambiguous case **(D)** occurring in a young female patient a few weeks postpartum. No intracoronary imaging was performed.

Because atherosclerotic disease is the most common cause of ACS, it is therefore also the most common differential diagnosis for SCAD. Rupture with fissuring can lead to contrast penetration of the atherosclerotic plaque core, sometimes giving an appearance akin to contrast penetration of a type 1 SCAD false lumen and even evolving into a localized plaque-associated dissection (Figure 4B). When present, these features are usually confined to the plaque location. Recanalized coronary thrombus from atherosclerotic plaque rupture can also sometimes generate multiple channels, giving an angiographic appearance similar to a type 1 SCAD. Longer atherosclerotic stenosis, particularly when affecting the mid-distal coronary vessel, may mimic classical type 2 appearances (Figure 4A). Type 3 SCAD by definition cannot be angiographically distinguished from atherosclerosis without intracoronary imaging (Figure 1C).

Particularly diagnostically challenging is the differential diagnosis between SCAD and highly localized rupture or erosion of noncalcified, lipid-rich atherosclerotic plaque leading to coronary thrombus formation. This is a cause of ACS in young patients and women (38,39) and can, like SCAD, be provoked by rigorous exercise (40). Furthermore, these lesions are often nonobstructive before the acute event (38,39,41) and, when managed without revascularization, may heal, leaving only minor residual stenosis (again like SCAD) (Figures 4B to 4D).

Three factors can be helpful in ambiguous cases in which the differential diagnosis lies between SCAD and atherosclerosis. First, the presence of substantial luminal thrombus (or evidence of thrombus embolization downstream of a stenosis) is highly suggestive of atherosclerotic disease. Second, intracoronary imaging such as intravascular ultrasound (IVUS) or OCT can elucidate the etiology (see the following discussion). Third, CTCA, particularly at convalescence, can help. The presence of coronary calcification or positive remodeled lipidemic plaque at the site of the culprit lesion is supportive of atherosclerosis, even when there is no overt stenotic disease (Figure 4C). Conversely, complete healing with no persisting vessel wall abnormality on CTCA at the site of the culprit lesion is supportive of SCAD.

### Coronary embolism

**Figure 5:**
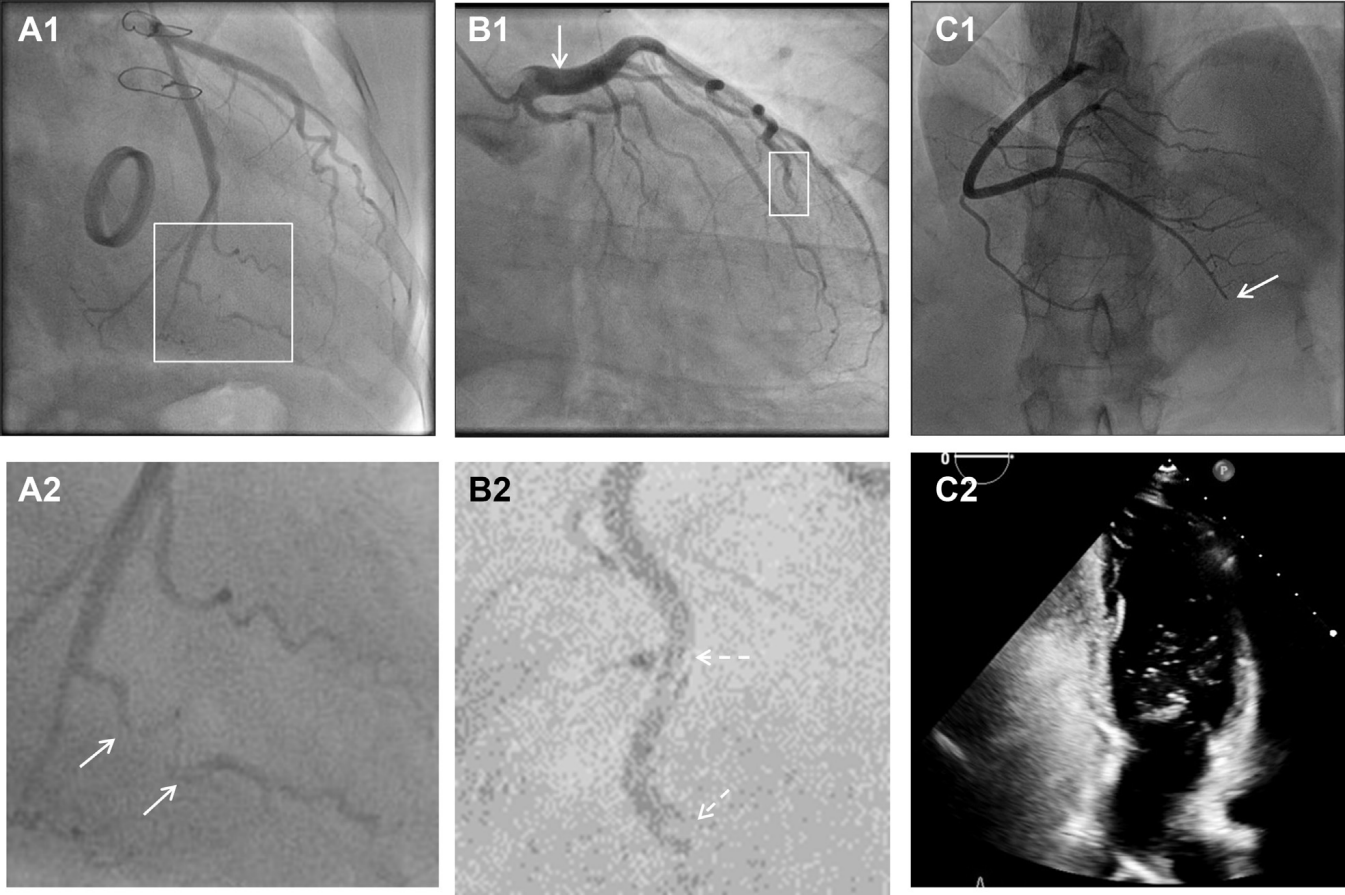
Coronary Embolism Versus SCAD **(A1, A2)** Narrowing of second marginal branch of circumflex coronary artery **(arrows)**. Appearances of spontaneous coronary artery dissection (SCAD) but note prosthetic valve **(A1)** as a potential source for coronary embolus. Distal occlusion of left anterior descending coronary artery (LAD) **(B1,B2)** with filling defect suggestive of type 1 SCAD but upstream ectasia **(arrows)** as a potential source for coronary thrombus. **(C1)** Distal right coronary artery occlusion with positive bubble study **(C2)**.

**Figure 6:**
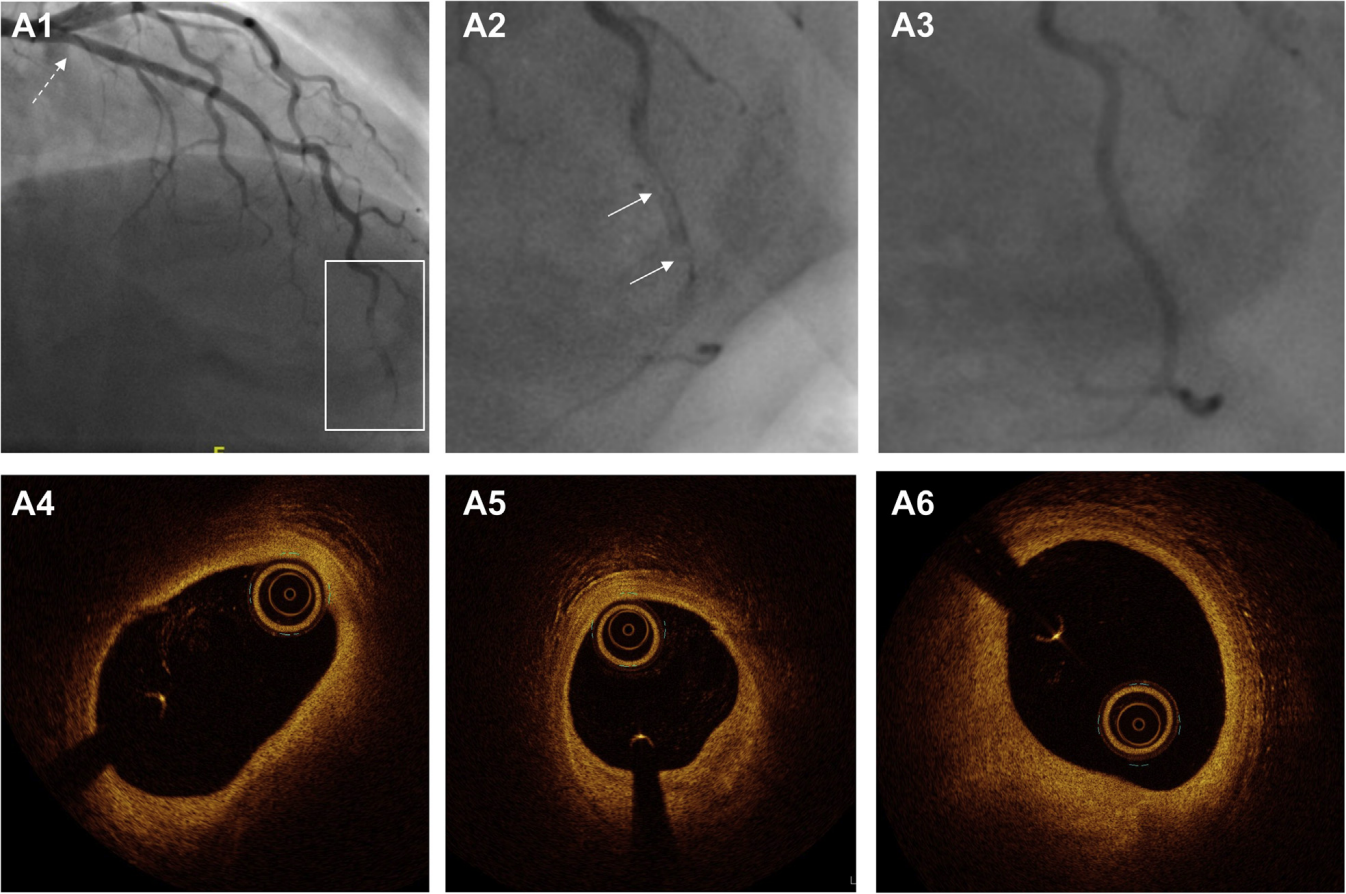
Atheroembolization Mimicking Spontaneous Coronary Artery Dissection Angiogram of a young female patient presenting with acute coronary syndrome **(A1)** showing abnormal apical left anterior descending coronary artery with dual-lumen appearance **(A2)**. Repeat angiography after 72-hour infusion of glycoprotein IIb/IIIa inhibitor shows complete resolution of these appearances in keeping with thrombus autolysis **(A3)**, and optical coherence tomographic appearances **(A4 to A6)** show extensive atheroma despite only mild angiographic disease **(A1)**.

Both SCAD (type 4) and coronary embolism can present with abrupt occlusion, often of a distal coronary territory (Figures 2A and 5). Contrast streaking around an embolic clot can also give the appearance of multiple channels, akin to type 1 SCAD (Figures 5A2, 5B2, and 6). Furthermore, clot resorption means that coronary embolus, like SCAD, will often resolve over time with restoration of normal coronary architecture. In some dissections, there will be a degree of narrowing upstream of the occlusion due to false lumen extension, which, when present, can aid in diagnosis (Figure 2A). Restoration of flow following wiring or limited percutaneous intervention may reveal more typical features of SCAD, such as a dual-lumen appearance (when not iatrogenic) or a long smooth intramural hematoma. With coronary embolus, a potential upstream source of thrombus may be evident, such as a metallic or rheumatic valve (Figure 5A), coronary ectasia (Figure 5B), or proximal atherosclerotic plaque (as a source of plaque-associated thrombus; Figure 6). There may also be truncation of multiple coronary branches, which is highly suggestive of an embolic source (although this must be carefully distinguished from multivessel SCAD, which occurs in about 15% of cases [9,42]). Sometimes additional investigations may be required to look for evidence of paradoxical embolism, cardiac source of embolus, or a predisposing hypercoagulable state (Figures 5C1 and 5C2).

### Takotsubo cardiomyopathy

**Figure 7:**
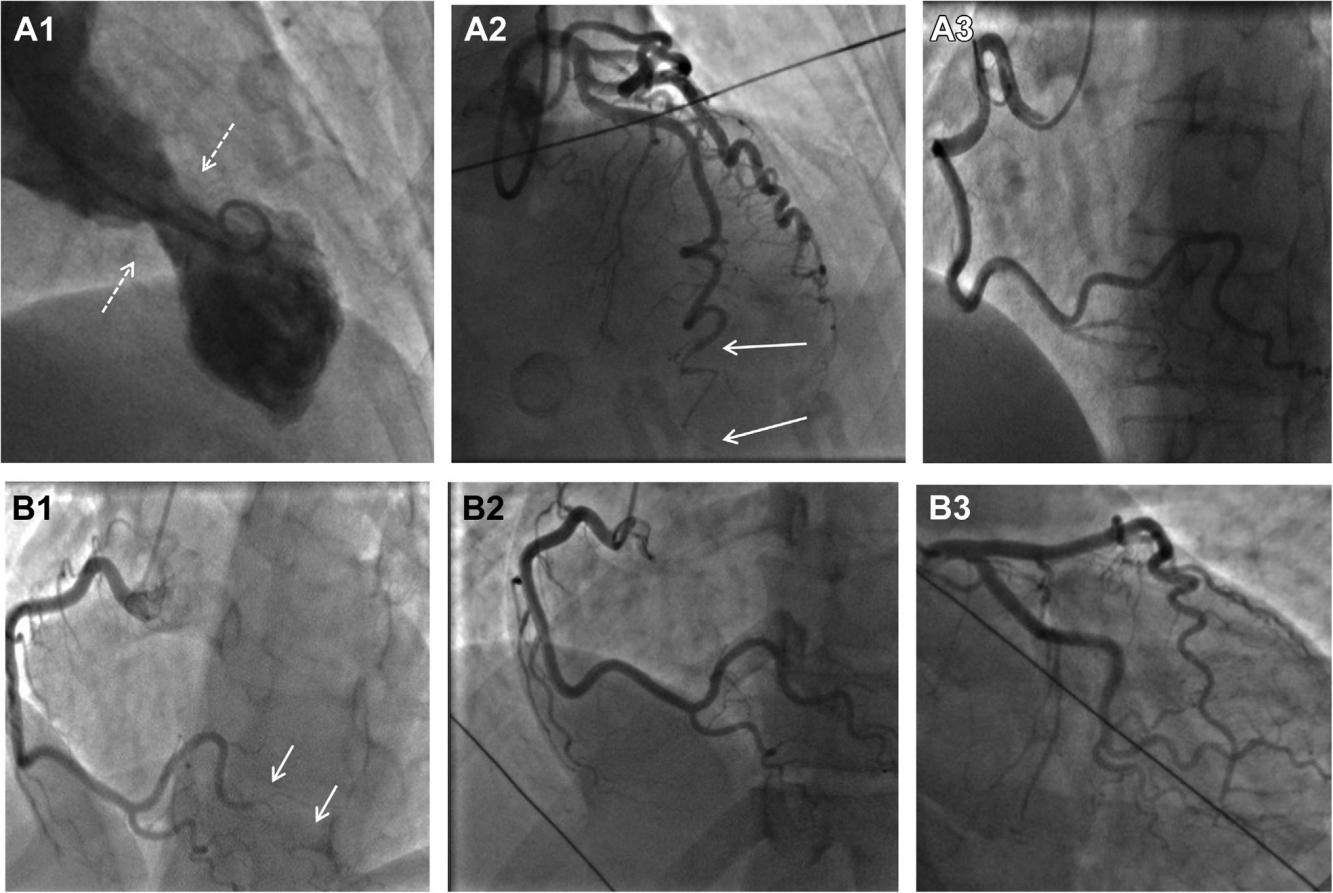
Takotsubo Cardiomyopathy or Myocardial Infarction With Nonobstructive Coronary Arteries Versus SCAD **(A1)** Typical apical ballooning with basal sparing on left ventricular angiography initially diagnosed as takotsubo cardiomyopathy. **(A2)** Careful examination of the apical segment of the left anterior descending coronary artery confirmed type 2b spontaneous coronary artery dissection (SCAD). **(A3)** Right coronary artery injection demonstrates extreme coronary tortuosity, a recognized angiographic feature in some patients with SCAD. **(B1)** Patient presenting with type 2A SCAD of the posterior left ventricular branch of the right coronary artery **(arrows)** managed conservatively. This patient subsequently presented with recurrent acute coronary syndrome, presumed clinically to be due to recurrent SCAD but with normal coronary arteries **(B2,B3)**, in keeping with myocardial infarction with nonobstructive coronary arteries.

Both SCAD and takotsubo cardiomyopathy affect predominantly women, with takotsubo affecting an older but overlapping population (43). Because SCAD has a predilection for more distal coronary territories and for the LAD, apical regional wall motion abnormalities akin to those seen in takotsubo cardiomyopathy are common (Figures 7A1 to 7A3, Supplemental Figure 2). Cardiac magnetic resonance imaging in convalescence may demonstrate a pattern of late gadolinium enhancement suggestive of infarction in a coronary territory, which might suggest SCAD. However, 40% of SCAD events resolve without evidence of lasting late gadolinium enhancement (44). Therefore, an apical regional wall motion abnormality that resolves at follow-up can occur with either diagnosis. Very careful assessment of the terminal branches of the LAD is therefore required on angiography. Some investigators have proposed a pathophysiological overlap between SCAD and takotsubo cardiomyopathy, but this remains unproved (45).

### Myocardial infarction in nonobstructed coronary arteries

Traditionally and somewhat confusingly given the “nonobstructive” definition, SCAD is included in many descriptions of the differential diagnosis of myocardial infarction in nonobstructed coronary arteries (Supplemental Figure 3). SCAD is frequently clearly obstructive, and many angiographically nonobstructive cases are likely to have been transiently obstructive (see the foregoing section on vasospasm). What is clear is that SCAD can affect very distal coronary territories, and careful review of invasive angiography is required in patients with ACS with apparently normal coronary arteries. Furthermore, it is now well recognized that SCAD is associated with a risk for recurrence (occurring in about 10% of patients over 3-year follow-up in one series [42]). We have noticed rare cases of recurrent ACS in patients with SCAD with no overt angiographic evidence of recurrent SCAD (Figures 7B1 to 7B3), suggesting that there may be some pathophysiological overlap between SCAD and myocardial infarction in nonobstructed coronary arteries.

### Iatrogenic dissection

**Figure 8:**
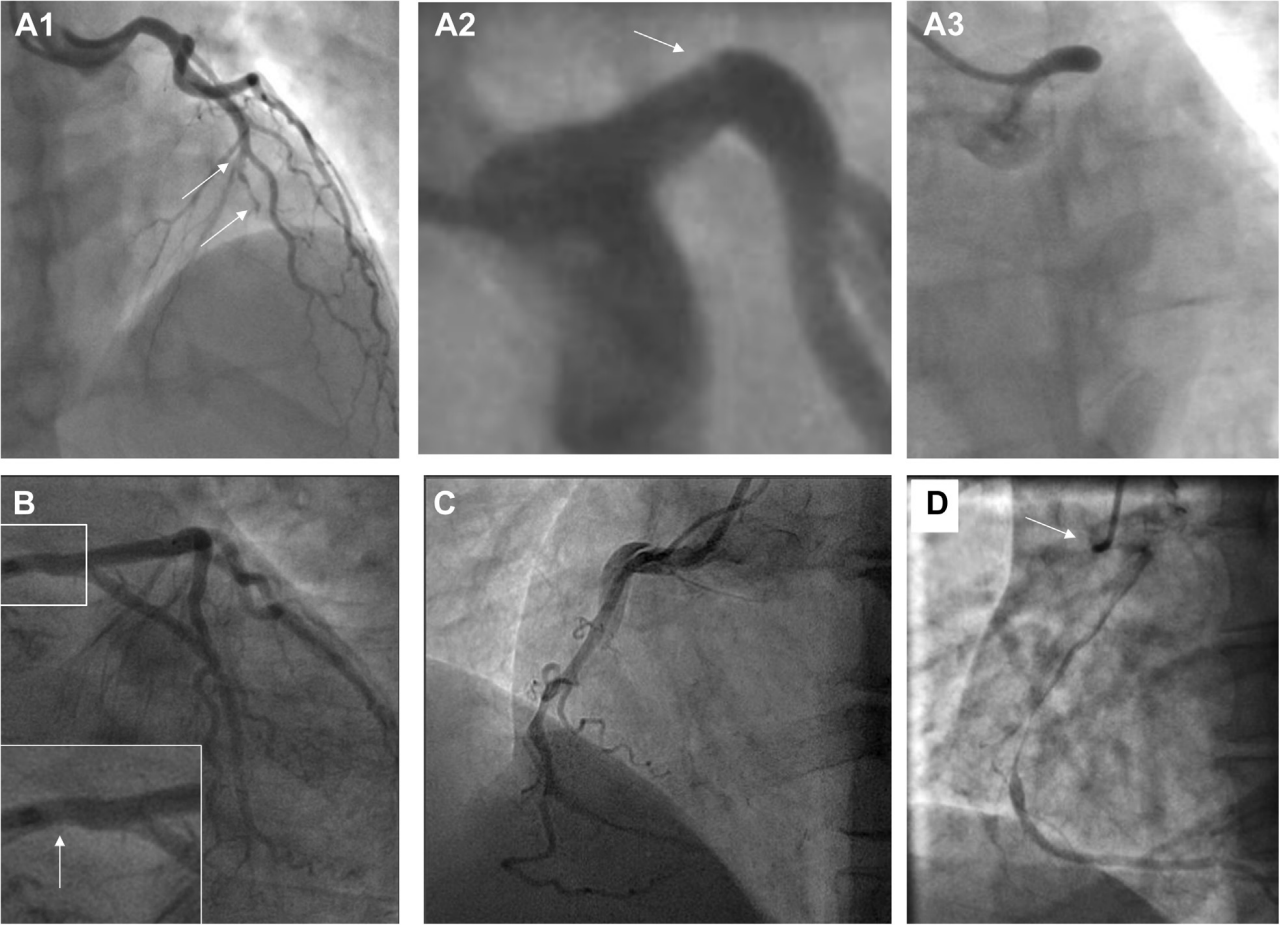
Iatrogenic Dissection Versus SCAD Spontaneous coronary artery dissection (SCAD) is associated with an increased risk for iatrogenic dissection **(A1 to A3)**. Initial angiography demonstrating type 4 SCAD in the mid left anterior descending coronary artery **(A1)**. During subsequent angiography, a linear filling defect is seen in the left mainstem **(A2)**, followed on the subsequent injection by complete occlusion **(A3)**. **(B)** In this case there is just a hint of a nonobstructive hematoma causing a minor stenosis **(arrow)** to suggest preexisting SCAD prior to extensive iatrogenic dissection (not shown) occurring on the next coronary injection. It can be difficult to be certain when dissection from the ostium is evident on the first contrast injection such as this right coronary artery injection **(C)**, unless there are preexisting inferior electrocardiographic changes or if a nonselective injection **(D, arrow)** confirms dissection before catheter engagement.

Patients with isolated iatrogenic dissection are not considered to have SCAD. However, it is well established that SCAD is associated with an increased risk for iatrogenic dissection (46) (Figures 8A1 to 8A3, Supplemental Figures 4A1 to 4A3). What is less clear is to what extent iatrogenic dissection in patients with ACS with otherwise normal coronary arteries occurs either because of preexisting proximal coronary SCAD (with the catheter penetrating the thin intimal-medial membrane and entering the false lumen on coronary intubation) or because of the same underlying vulnerability (Figures 8B and 8C). In some cases, contrast injection before coronary intubation can demonstrate a preexisting proximal hematoma, confirming SCAD prior to iatrogenic dissection (Figure 8D). In other cases, SCAD may be demonstrated elsewhere in the coronary tree (Figures 8A1 to 8A3). Rarely, dissection may arise from trauma or even from an active fixation pacing wire (Supplemental Figures 4B1 to 4B3). Dissections occurring in this context would not be considered as SCAD.

### Oddities: Is this even SCAD?

There are some angiographic appearances that appear different from classical SCAD and may not be part of the same clinical syndrome. These include those with a chronic dual-lumen or multichannel appearance in which the differential seems to lie between recanalization of an occlusion and some form of chronic dissection (Supplemental Figure 5). Likewise, it is unclear if ectasia-associated dissection is part of the SCAD spectrum of coronary arteriopathies or is pathophysiologically distinct (Supplemental Figure 6).

## Intracoronary Imaging: Use and Limitations

Although invasive coronary angiography provides diagnostic images for most patients with SCAD, there remain cases in which angiography alone leaves uncertainty. In these cases, intracoronary imaging is frequently helpful but carries a small added procedural risk in the already fragile arteries in SCAD. The largest published coronary imaging series confirm a small number of complications (5 of 63 cases) directly attributable to imaging, all of which were managed either conservatively or with PCI and without adverse sequelae (5). For this reason, routine imaging is not advocated. However, the paramount importance of accurate diagnosis justifies imaging in most cases in which it is feasible, and angiography alone leaves diagnostic doubt.

**Figure 9:**
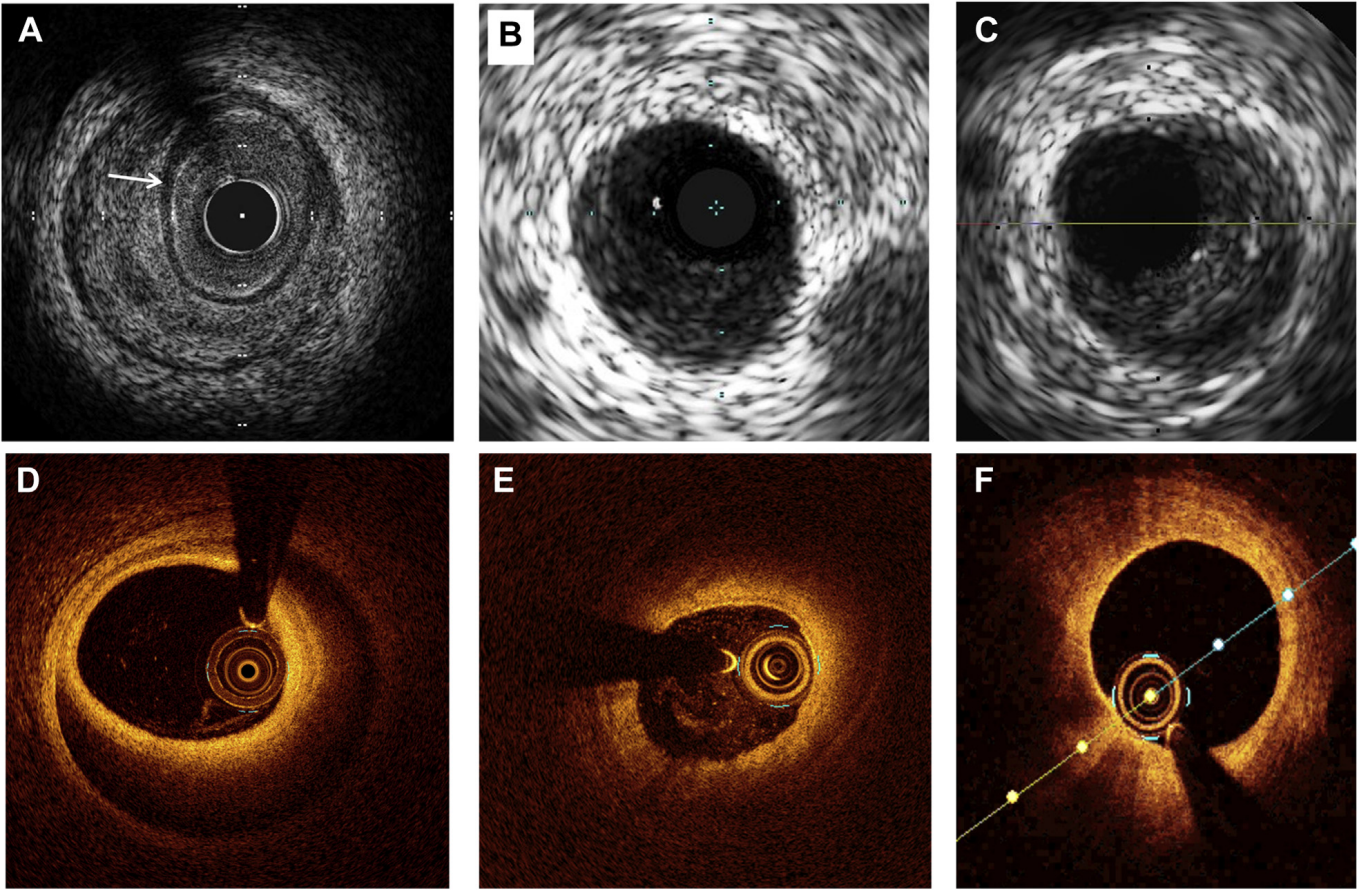
IVUS and OCT Images Intravascular ultrasound (IVUS) image of spontaneous coronary artery dissection (SCAD) **(A)** showing classical white-black-white appearance of the intimal-medial membrane, which is pathognomonic of SCAD **(arrow)**. When not present, SCAD **(B)** can be very difficult to distinguish from lipidemic atherosclerotic plaque **(C)** on IVUS. Careful scrutiny of the entire pull back length may be required in such cases. Optical coherence tomographic (OCT) imaging of SCAD **(D)** clearly showing the intimal-medial membrane, false lumen, and external elastic lamina. Sometimes light penetration to the external elastic lamina may be limited **(E)**, but with careful inspection OCT imaging can usually be distinguished from lipidemic atherosclerotic plaque **(F)**.

IVUS has the theoretical advantages over OCT of greater depth penetration and avoidance of the need for blood clearance by high-pressure injection for imaging. Typical IVUS features of SCAD are described (Figure 9A). In particular, the triple band (white-black-white) of the intimal-medial membrane is pathognomonic of SCAD. However, imaging with IVUS is marred by the inherently lower spatial resolution, and because of this, it can be challenging with IVUS to distinguish SCAD from lipid-rich atheroma (a key differential diagnosis) (Figures 9B and 9C).

For this reason, when intracoronary imaging is required for diagnosis, OCT with its much higher spatial resolution is generally better (Figures 9D to 9F). Theoretical risks for dissection extension with contrast injection have not been borne out in described series to date, although caution in very proximal type 1 dissections may be sensible, and it is not usually necessary to image the entire SCAD length. It is worth remembering that light penetration of the false lumen is variable, particularly in type 2 cases in which contrast has not penetrated into the false lumen (Figure 9E) or luminal blood clearance with contrast injection is difficult. Careful image review for the typical features of SCAD is therefore needed, but it is usually possible to distinguish SCAD from lipid-rich atheroma with OCT (Figure 9F).

In some situations, however, intracoronary imaging in patients with SCAD may not be feasible, particularly in cases with severe tortuosity or when SCAD occurs in distal, small-caliber arteries.

## After Angiography

When diagnostic uncertainty remains after angiography and intracoronary imaging, further investigations may provide additional diagnostic insights. For example, early cardiac magnetic resonance imaging may help identify features suggestive of other nonischemic causes of myocardial injury, such as myocarditis (39).

Series that have included angiographic follow-up have shown that SCAD heals, with restoration of a normal coronary architecture, in almost all cases. Given the known risk for iatrogenic dissection in acute SCAD (46), routine follow-up invasive angiography is not recommended (1,2). The role of routine follow-up CTCA remains unclear, although it may have a place in confirming healing of SCAD affecting the proximal coronary territories, for which the spatial resolution of CTCA is more favorable. However, in ambiguous cases in which the diagnosis remains uncertain, follow-up angiography, whether invasive or by CTCA, can sometimes be helpful to establish the etiology of ACS. A demonstration of complete healing is consistent with SCAD and can be helpful for some cases (although coronary thrombus including emboli may also similarly heal, albeit on a shorter time scale) (Figure 6). Persisting stenosis, even mild, and presence of coronary calcium or positively remodeled atherosclerotic plaque (seen best with CTCA) can refute a diagnosis of SCAD in favor of an atherosclerotic event or prompt percutaneous intervention when deemed appropriate. When repeat coronary assessment is contemplated, it is important to allow sufficient time for healing. SCAD studies with angiographic follow-up suggest that most will have healed by 1 month, but when imaging is driven by diagnostic considerations rather than symptoms, it may be sensible to wait up to 6 months (9,47,48).

SCAD has a strong association with extracoronary arteriopathies, particularly FMD, which occurs in at least one third of patients (8,49). Brain-to-pelvis cross-sectional imaging to screen for coexistent aneurysm, extracoronary dissection, or FMD is currently recommended (1,2). Some centers also advocate fluoroscopic angiography of the renal arteries at the time of coronary angiography. The presence of FMD in a patient whose angiogram is nondiagnostic is supportive of a SCAD diagnosis, as FMD appears to be uncommon in the general population (50). However, atherosclerotic ACS has also been described in the context of FMD (51).

## Conclusion and Suggested Approach

Most SCAD can be confidently diagnosed angiographically, and for these patients further investigations beyond extracoronary arteriopathy screening and an assessment of left ventricular function are unnecessary. However, some cases are challenging, and in these patients further investigations may help establish or refute a firm diagnosis of SCAD. This can be critical to determine the best long-term management. A suggested approach to investigations for different angiographic scenarios is shown in the Central Illustration.

**Central Illustration:**
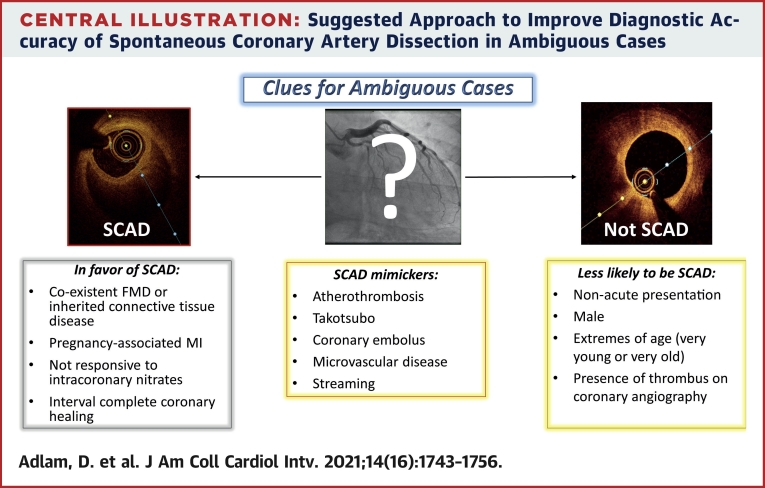
Suggested Approach to Improve Diagnostic Accuracy of Spontaneous Coronary Artery Dissection in Ambiguous Cases FMD = fibromuscular dysplasia; SCAD = spontaneous coronary artery dissection.

## Funding Support and Author Disclosures

This SCAD research study was supported by BeatSCAD, the British Heart Foundation (PG/13/96/30608), the National Institute for Health Research rare disease translational collaboration, the Leicester NIHR Biomedical Research Centre, and SCAD Research. Dr Moss receives funding support from the British Heart Foundation (AA/18/3/34220). Dr Adlam has received research funding from Abbott Vascular for a clinical research fellow and from AstraZeneca for SCAD genetics research and unrelated research; and has undertaken consultancy with General Electric to support general research funds. All other authors have reported that they have no relationships relevant to the contents of this paper to disclose.

## References

[1] Adlam D., Alfonso F., Maas A., Vrints C., Writing Committee (2018). European Society of Cardiology, acute cardiovascular care association, SCAD study group: a position paper on spontaneous coronary artery dissection. Eur Heart J.

[2] Hayes S.N., Kim E.S.H., Saw J. (2018). Spontaneous coronary artery dissection: current state of the science: a scientific statement from the American Heart Association. Circulation.

[3] Hayes S.N., Tweet M.S., Adlam D. (2020). Spontaneous coronary artery dissection: JACC state-of-the-art review. J Am Coll Cardiol.

[4] Kim E.S.H. (2020). Spontaneous coronary-artery dissection. N Engl J Med.

[5] Jackson R., Al-Hussaini A., Joseph S. (2019). Spontaneous coronary artery dissection: pathophysiological insights from optical coherence tomography. J Am Coll Cardiol Img.

[6] Waterbury T.M., Tweet M.S., Hayes S.N. (2018). Early natural history of spontaneous coronary artery dissection. Circ Cardiovasc Interv.

[7] Phan D., Clare R., Duan L.W., Kim C., Moore N., Lee M.S. (2021). Characteristics and outcomes of patients with spontaneous coronary artery dissection who suffered sudden cardiac arrest. J Interv Card Electr.

[8] Saw J., Aymong E., Sedlak T. (2014). Spontaneous coronary artery dissection: association with predisposing arteriopathies and precipitating stressors and cardiovascular outcomes. Circ Cardiovasc Interv.

[9] Tweet M.S., Eleid M.F., Best P.J. (2014). Spontaneous coronary artery dissection: revascularization versus conservative therapy. Circ Cardiovasc Interv.

[10] Russo V., Marrozzini C., Zompatori M. (2013). Spontaneous coronary artery dissection: role of coronary CT angiography. Heart.

[11] Lindor R.A., Tweet M.S., Goyal K.A. (2017). Emergency department presentation of patients with spontaneous coronary artery dissection. J Emerg Med.

[12] Saw J., Starovoytov A., Humphries K. (2019). Canadian spontaneous coronary artery dissection cohort study: in-hospital and 30-day outcomes. Eur Heart J.

[13] Sharma S., Kaadan M.I., Duran J.M. (2019). Risk factors, imaging findings, and sex differences in spontaneous coronary artery dissection. Am J Cardiol.

[14] Nakashima T., Noguchi T., Haruta S. (2016). Prognostic impact of spontaneous coronary artery dissection in young female patients with acute myocardial infarction: a report from the Angina Pectoris-Myocardial Infarction Multicenter Investigators in Japan. Int J Cardiol.

[15] Meng P.N., Xu C., You W. (2017). Spontaneous coronary artery dissection as a cause of acute myocardial infarction in young female population: a single-center study. Chin Med J (Engl).

[16] Saw J., Aymong E., Mancini G.B., Sedlak T., Starovoytov A., Ricci D. (2014). Nonatherosclerotic coronary artery disease in young women. Can J Cardiol.

[17] Elkayam U., Jalnapurkar S., Barakkat M.N. (2014). Pregnancy-associated acute myocardial infarction: a review of contemporary experience in 150 cases between 2006 and 2011. Circulation.

[18] Smilowitz N.R., Gupta N., Guo Y. (2018). Acute myocardial infarction during pregnancy and the puerperium in the United States. Mayo Clin Proc.

[19] Henkin S., Negrotto S.M., Tweet M.S. (2016). Spontaneous coronary artery dissection and its association with heritable connective tissue disorders. Heart.

[20] Kaadan M.I., MacDonald C., Ponzini F. (2018). Prospective cardiovascular genetics evaluation in spontaneous coronary artery dissection. Circ Genom Precis Med.

[21] Carss K.J., Baranowska A.A., Armisen J. (2020). Spontaneous coronary artery dissection: insights on rare genetic variation from genome sequencing. Circ Genom Precis Med.

[22] Luong C., Starovoytov A., Heydari M., Sedlak T., Aymong E., Saw J. (2017). Clinical presentation of patients with spontaneous coronary artery dissection. Catheter Cardiovasc Interv.

[23] Saw J., Ricci D., Starovoytov A., Fox R., Buller C.E. (2013). Spontaneous coronary artery dissection: prevalence of predisposing conditions including fibromuscular dysplasia in a tertiary center cohort. J Am Coll Cardiol Intv.

[24] Ghadri J.R., Wittstein I.S., Prasad A. (2018). International expert consensus document on takotsubo syndrome (part I): clinical characteristics, diagnostic criteria, and pathophysiology. Eur Heart J.

[25] Franklin B.A., Thompson P.D., Al-Zaiti S.S. (2020). Exercise-related acute cardiovascular events and potential deleterious adaptations following long-term exercise training: placing the risks into perspective-an update: a scientific statement from the American Heart Association. Circulation.

[26] Motreff P., Malcles G., Combaret N. (2017). How and when to suspect spontaneous coronary artery dissection: novel insights from a single-centre series on prevalence and angiographic appearance. EuroIntervention.

[27] Yip A., Saw J. (2015). Spontaneous coronary artery dissection—a review. Cardiovasc Diagn Ther.

[28] Mori R, Macaya F, Giacobbe F, et al. Clinical outcomes by angiographic type of spontaneous coronary artery dissection. *EuroIntervention* 2021 Mar 2 [E-pub ahead of print]. 10.4244/EIJ-D-20-01275 PMC9724881 33650491

[29] Eleid M.F., Guddeti R.R., Tweet M.S. (2014). Coronary artery tortuosity in spontaneous coronary artery dissection: angiographic characteristics and clinical implications. Circ Cardiovasc Interv.

[30] Combaret N, Gerbaud E, Derimay F, et al. National French Registry of Spontaneous Coronary Artery Dissections: prevalence of fibromuscular dysplasia and genetic analyses. *EuroIntervention* 2020 Dec 15 [E-pub ahead of print]. 10.4244/EIJ-D-20-01046 PMC9725012 33319763

[31] Garcia-Guimaraes M., Bastante T., Antuna P. (2020). Spontaneous coronary artery dissection: mechanisms, diagnosis and management. Eur Cardiol.

[32] Al-Hussaini A., Adlam D. (2017). Spontaneous coronary artery dissection. Heart.

[33] Adlam D., Olson T.M., Combaret N. (2019). Association of the PHACTR1/EDN1 genetic locus with spontaneous coronary artery dissection. J Am Coll Cardiol.

[34] Saw J., Yang M.-L., Trinder M. (2020). Chromosome 1q21.2 and additional loci influence risk of spontaneous coronary artery dissection and myocardial infarction. Nat Commun.

[35] Turley T.N., O’Byrne M.M., Kosel M.L. (2020). Identification of susceptibility loci for spontaneous coronary artery dissection. JAMA Cardiol.

[36] Margaritis M, Saini F, Baranowska-Clarke AA, et al. Vascular histopathology and connective tissue ultrastructure in spontaneous coronary artery dissection: pathophysiological and clinical implications. *Cardiovasc Res**.* 2021 May 28 [E-pub ahead of print]. 10.1093/cvr/cvab183 PMC9215198 34048532

[37] Saw J., Bezerra H., Gornik H.L., Machan L., Mancini G.B. (2016). Angiographic and intracoronary manifestations of coronary fibromuscular dysplasia. Circulation.

[38] DeFilippis E.M., Collins B.L., Singh A. (2020). Women who experience a myocardial infarction at a young age have worse outcomes compared with men: the Mass General Brigham YOUNG-MI registry. Eur Heart J.

[39] Reynolds H.R., Maehara A., Kwong R.Y. (2021). Coronary optical coherence tomography and cardiac magnetic resonance imaging to determine underlying causes of myocardial infarction with nonobstructive coronary arteries in women. Circulation.

[40] Mas-Llado C., Maristany J., Gomez-Lara J. (2020). Optical coherence tomography for the diagnosis of exercise-related acute cardiovascular events and inconclusive coronary angiography. J Interv Cardiol.

[41] Jia H., Dai J., Hou J. (2017). Effective anti-thrombotic therapy without stenting: intravascular optical coherence tomography-based management in plaque erosion (the EROSION study). Eur Heart J.

[42] Saw J., Humphries K., Aymong E. (2017). Spontaneous coronary artery dissection: clinical outcomes and risk of recurrence. J Am Coll Cardiol.

[43] Salamanca J, Garcia-Guimaraes M, Camacho-Freire SJ, et al. Spontaneous coronary artery dissection and Takotsubo syndrome: comparison of baseline clinical and angiographic characteristics and in-hospital outcomes. *Coron Artery Dis* 2020 Nov 12 [E-pub ahead of print]. 10.1097/MCA.0000000000000984 33186146

[44] Al-Hussaini A., Abdelaty A., Gulsin G.S. (2020). Chronic infarct size after spontaneous coronary artery dissection: implications for pathophysiology and clinical management. Eur Heart J.

[45] S Y.H., Themudo R., Maret E. (2017). Spontaneous coronary artery dissection and takotsubo syndrome: the chicken or the egg causality dilemma. Catheter Cardiovasc Interv.

[46] Prakash R., Starovoytov A., Heydari M., Mancini G.B., Saw J. (2016). Catheter-induced iatrogenic coronary artery dissection in patients with spontaneous coronary artery dissection. J Am Coll Cardiol Intv.

[47] Rogowski S., Maeder M.T., Weilenmann D. (2017). Spontaneous coronary artery dissection: angiographic follow-up and long-term clinical outcome in a predominantly medically treated population. Catheter Cardiovasc Interv.

[48] Roura G., Ariza-Sole A., Rodriguez-Caballero I.F. (2016). Noninvasive follow-up of patients with spontaneous coronary artery dissection with CT angiography. J Am Coll Cardiol Img.

[49] Prasad M., Tweet M.S., Hayes S.N. (2015). Prevalence of extracoronary vascular abnormalities and fibromuscular dysplasia in patients with spontaneous coronary artery dissection. Am J Cardiol.

[50] Hendricks N.J., Matsumoto A.H., Angle J.F. (2014). Is fibromuscular dysplasia underdiagnosed? A comparison of the prevalence of FMD seen in CORAL trial participants versus a single institution population of renal donor candidates. Vasc Med.

[51] Tweet M.S., Akhtar N.J., Hayes S.N., Best P.J., Gulati R., Araoz P.A. (2019). Spontaneous coronary artery dissection: Acute findings on coronary computed tomography angiography. Eur Heart J Acute Cardiovasc Care.

