# Supplementary material for: Spontaneous Coronary Artery Dissection: Pitfalls of Angiographic Diagnosis and an Approach to Ambiguous Cases
Source: JACC Cardiovasc Interv. 2021 Aug 23;14(16):1743–56. doi: 10.1016/j.jcin.2021.06.027 (PMC8383825; doi:10.1016/j.jcin.2021.06.027)
Supplement: Supplemental Data [file mmc1.docx]

## Supplemental Material

**Spontaneous coronary artery dissection: pitfalls of angiographic diagnosis and an approach to ambiguous cases**

David Adlam^a^, MD, PhD; Marysia S. Tweet^b^, MD; Rajiv Gulati^b^ MD, PHD; Deevia Kotecha MB BS^a^; Praveen Rao^a^, MD; Alistair Moss^a^, MD; Sharonne N. Hayes^c^, MD

^a^ Department of Cardiovascular Sciences, and NIHR Leicester Biomedical Research Centre, University of Leicester, UK

^b^Department of Cardiovascular Medicine, Mayo Clinic, Rochester Minnesota, USA

**
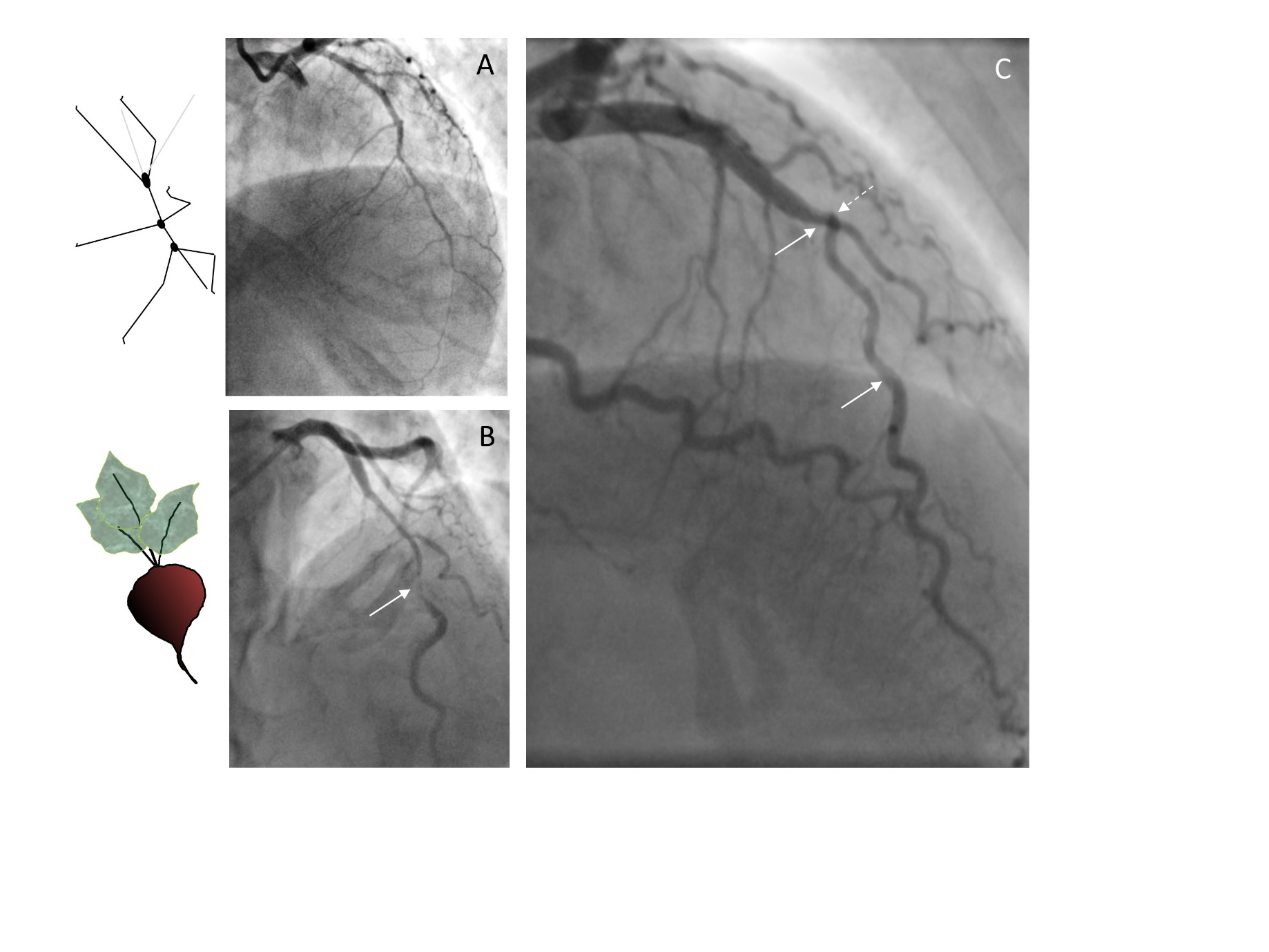
**

**Supplemental Figure1. Other SCAD angiographic aide-memoire.** The ‘stick insect’ and the ‘radish’ – proposed by Motreff et al. (1,2) help recognition of SCAD. The ‘stick insect’ represents the long segments of stenosis with patent side-branches seen in some SCAD cases (A). The ‘radish’ represents the propensity for a severe near-occlusive narrowing at the distal extent of SCAD (B). The ‘broken-line’ is a feature of SCAD in tortuous segments (C – white arrows) proposed by Alfonso et al. (3) where the haematoma either leads to straightening of the arterial curves and may cause localised kinking (dotted arrow).


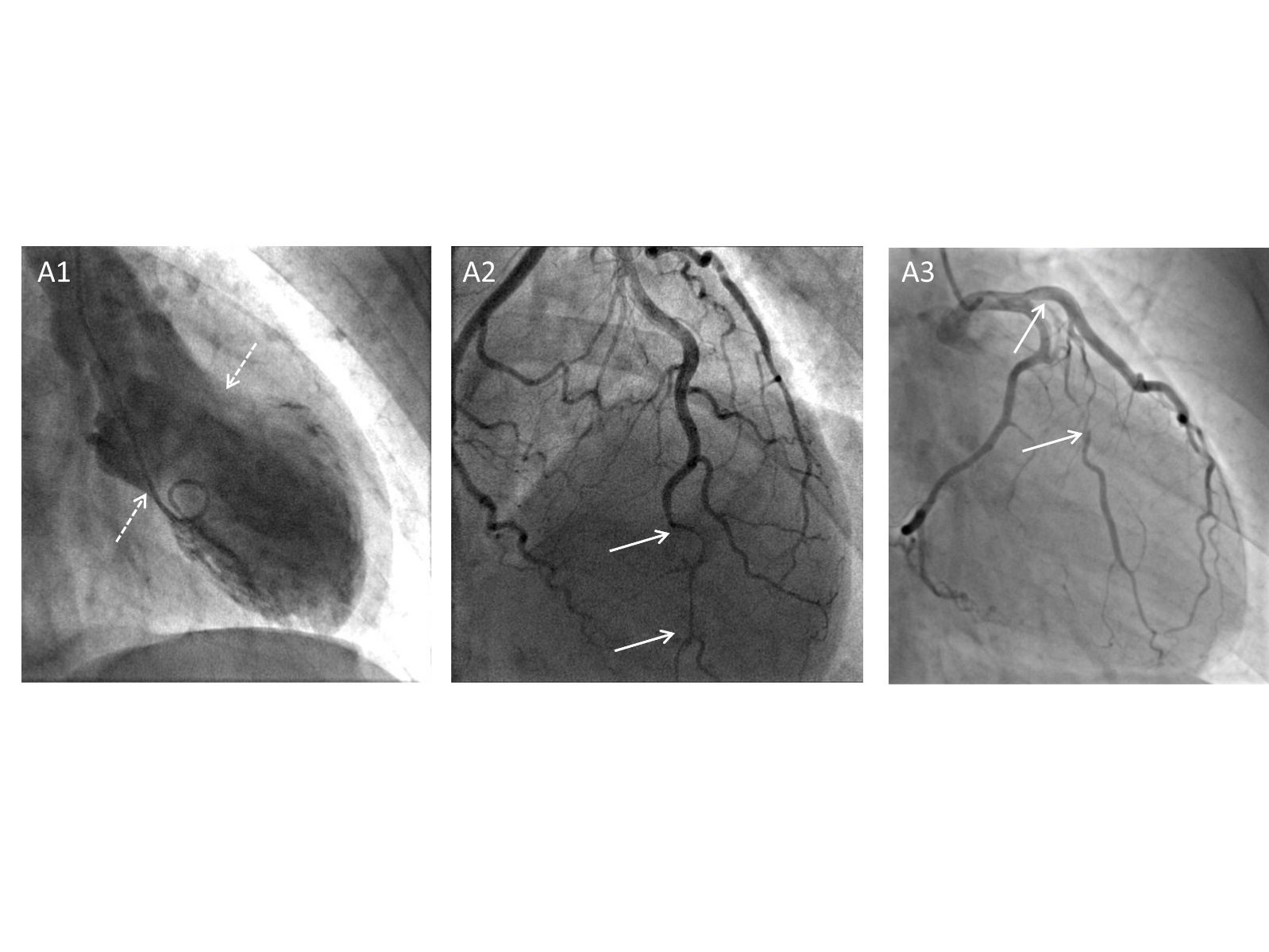


**Supplemental Figure 2: SCAD mimicking Takotsubo cardiomyopathy:** (A1) Typical apical ballooning with basal sparing on left ventricular angiography initially diagnosed as Takotsubo. Years later the patient represented with Type 2A SCAD of a large intermediate vessel (A3). Review of the original angiogram confirmed a missed Type 2B SCAD of the apical LAD (A2).


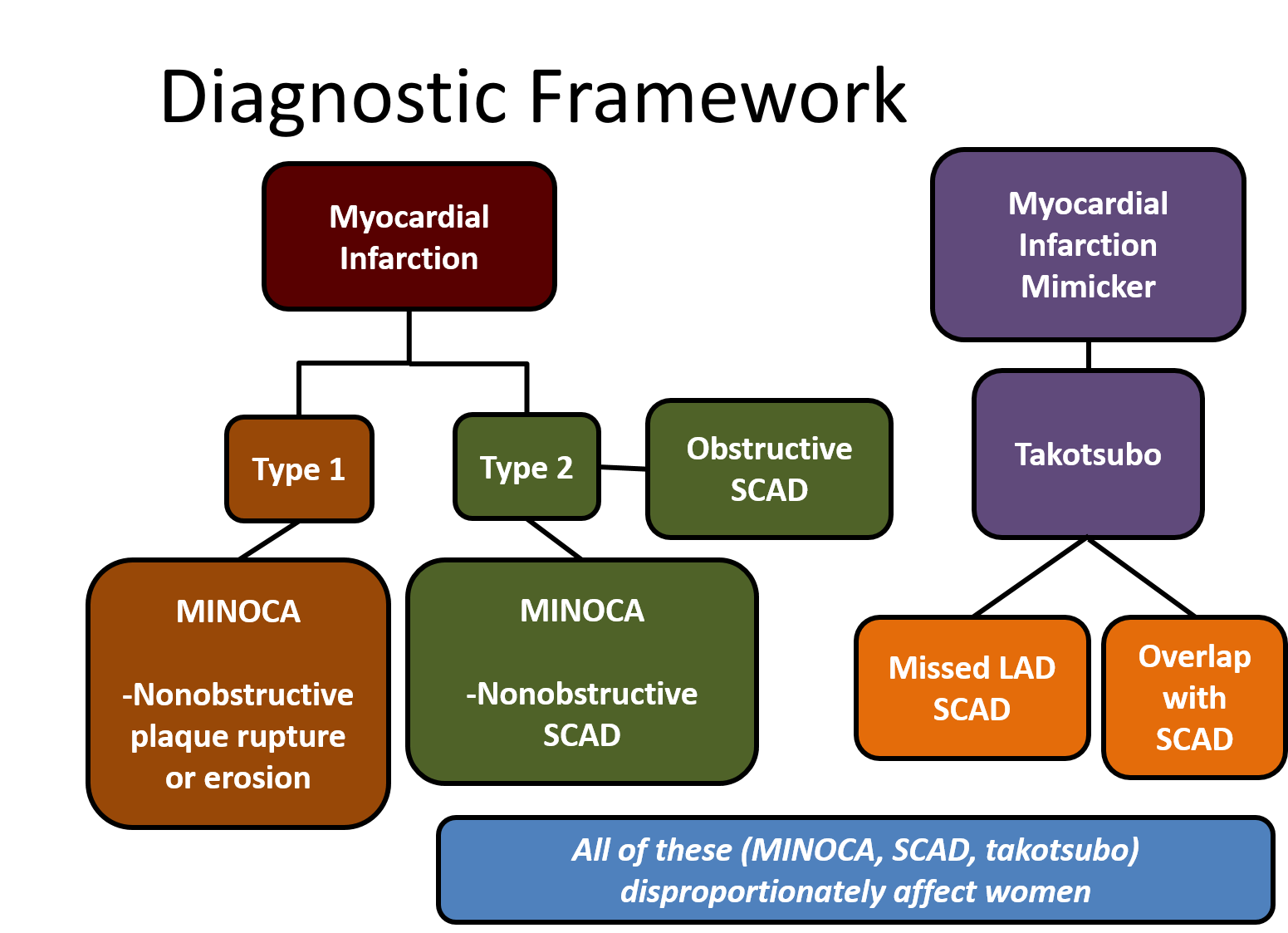


**Supplemental Figure 3: Diagnostic Framework for SCAD.** SCAD is traditionally regarded as an exemplar of Type 2 AMI despite being frequently obstructive. When non-obstructive SCAD is a differential diagnosis for MINOCA. Apical LAD SCAD may also mimic Takotsubo cardiomyopathy.


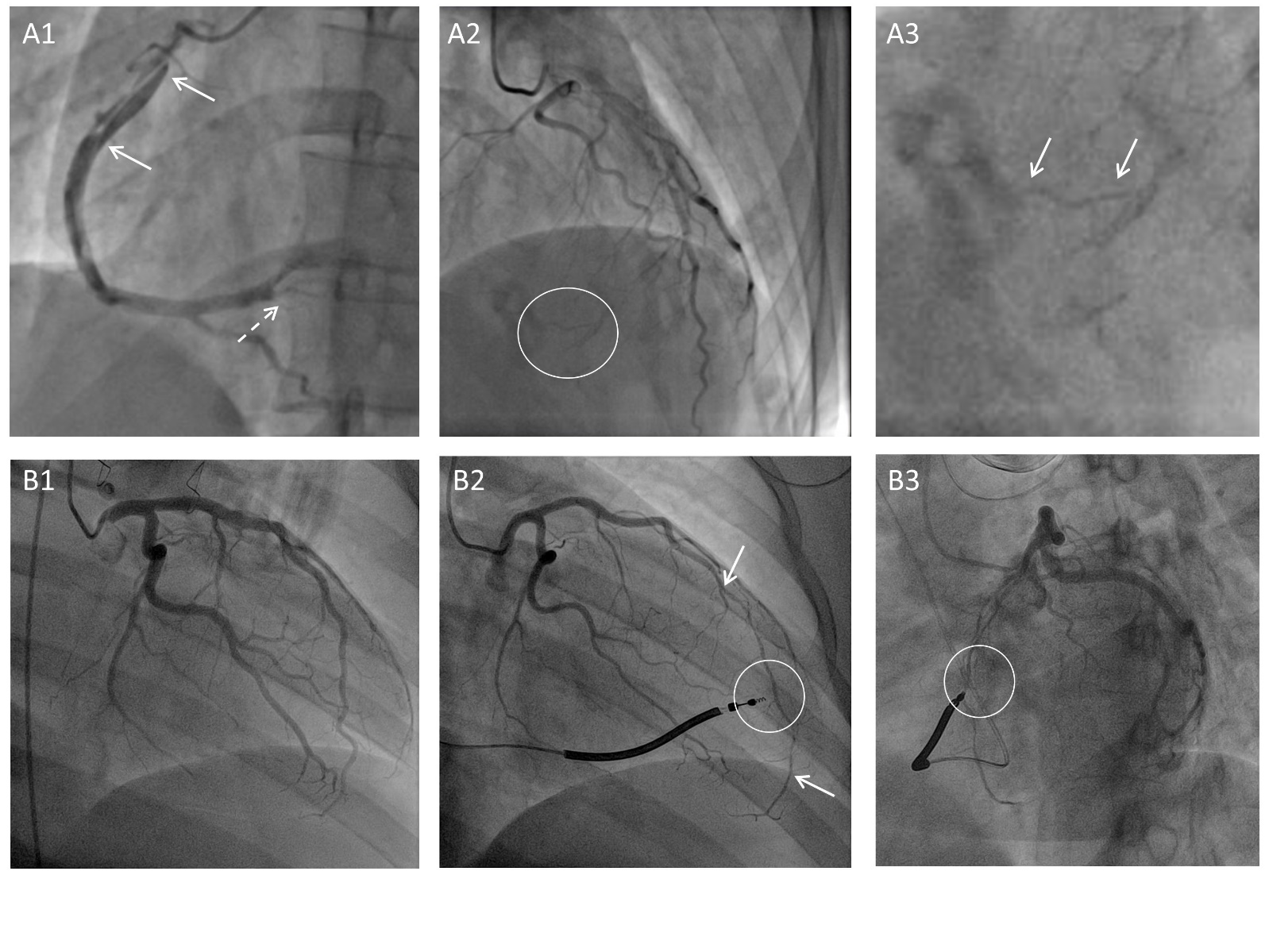


**Supplemental Figure 4: Iatrogenic dissection versus SCAD.** (A1) Apparent catheter induced dissection of proximal the RCA (solid arrows). Closer inspection revealed an occlusive dissection of the posterior descending branch (dotted arrows) in keeping with the clinical and ECG presentation. The left coronary injections taken before RCA intubation also show evidence of collaterals to the distal RCA (A2 circled and A3 arrows). Therefore likely iatrogenic dissection complicating SCAD. (B1) Patient presenting with cardiac arrest angiographically normal coronary arteries and a dilated cardiomyopathy was readmitted after ICD implantation with an anterior MI. Subsequent Angiography showed dissection of the mid-distal LAD (B2 & B3) in very close proximity to the active fixation ICD lead (circled).


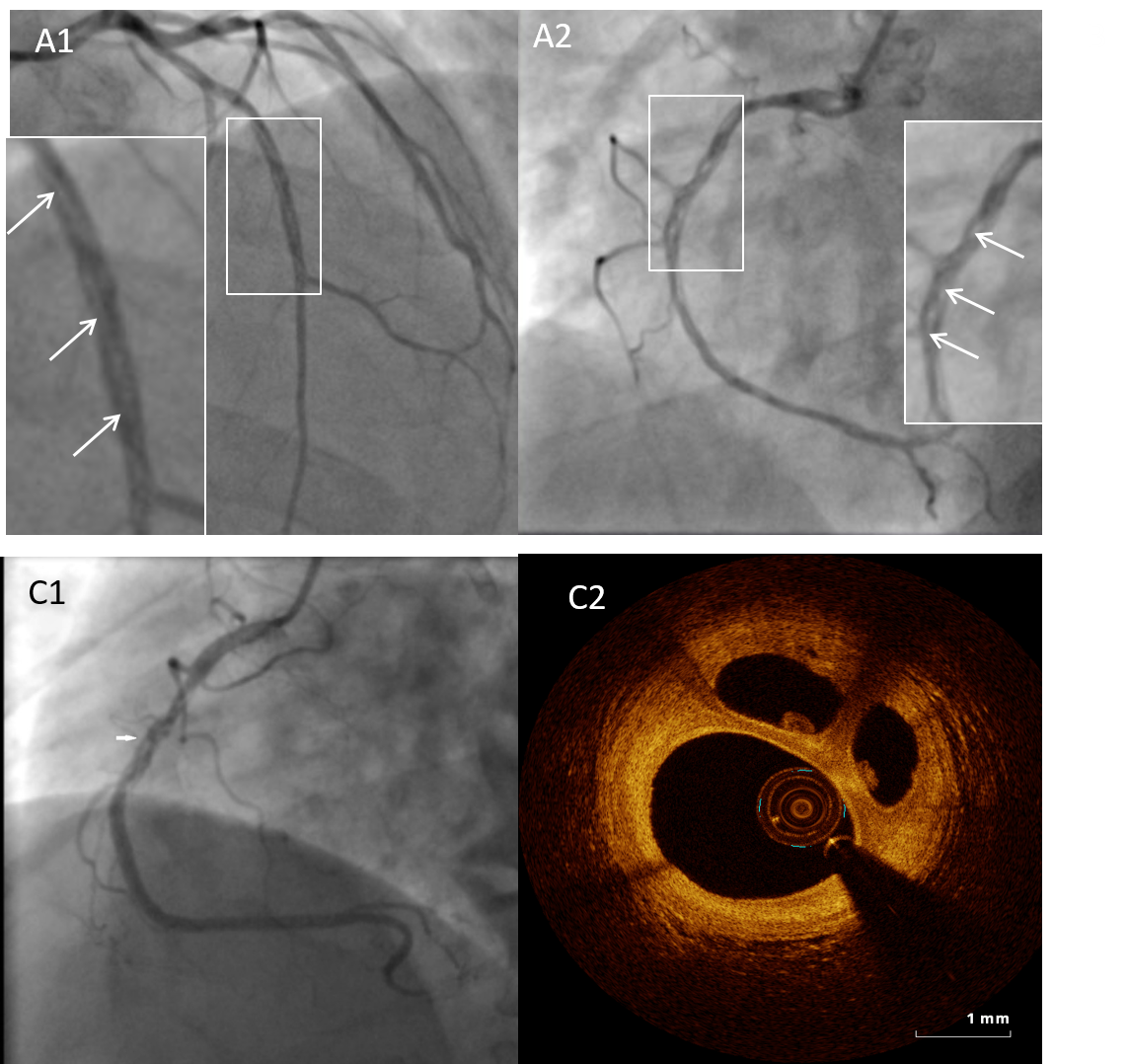


**Supplemental Figure 5. Coronary segments with multi-channel appearance.** A1 & A2 affecting multiple vessels in the same patient with stable appearances over many years. C1 affecting the right coronary artery with OCT image C2 indicating multiple channels separated by septae. It is unclear if these appearance represent chronic dissections, recanalised coronary occlusions or another process.


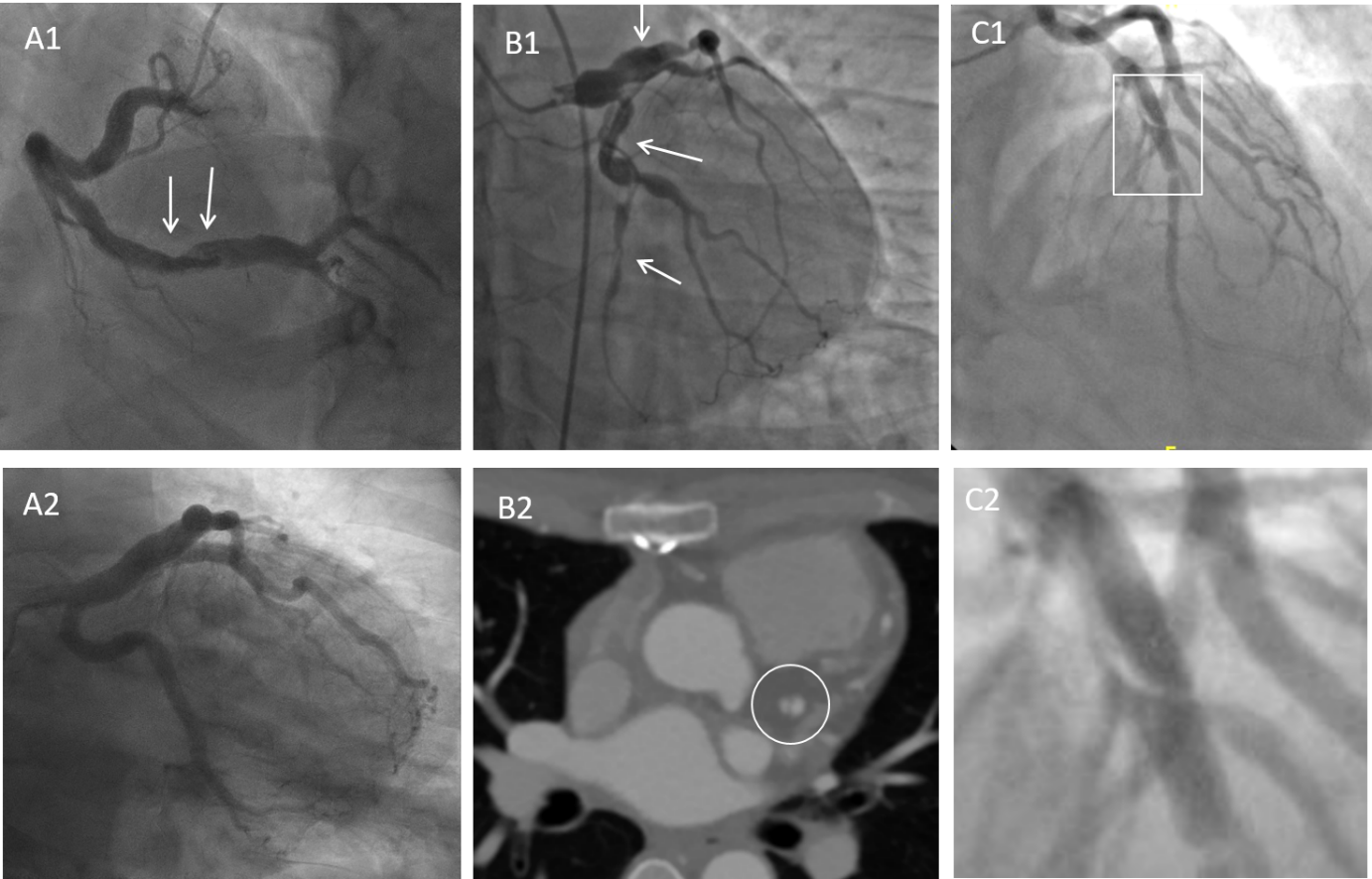


**Supplemental Figure 6: Coronary Ectasia and SCAD:** Coronary ectasia may alter coronary flow leading to streaming patterns mimicking Type 1 SCAD (arrows) (A1). This patient had distal thrombus in the RPDA. (A2) The left coronary artery is also diffusely ectatic. Coronary ectasia may also be associated with definite dissection (B & C); Multi-vessel dissection in an ectatic LAD and left circumflex coronary arteries (B1). Coronary CTA showed persistent dissections (B2). LAD ectasia-associated dissection SCAD (C1 and enlarged C2). It is not clear if this represents the same pathophysiological process as SCAD.

**Supplemental References**

1. Combaret N, Gerbaud E, Derimay F et al. National French Registry of Spontaneous Coronary Artery Dissections: Prevalence of Fibromuscular Dysplasia and Genetic Analyses. EuroIntervention : journal of EuroPCR in collaboration with the Working Group on Interventional Cardiology of the European Society of Cardiology 2020.

2. Motreff P, Malcles G, Combaret N et al. How and when to suspect spontaneous coronary artery dissection: novel insights from a single-centre series on prevalence and angiographic appearance. EuroIntervention : journal of EuroPCR in collaboration with the Working Group on Interventional Cardiology of the European Society of Cardiology 2017;12:e2236-e2243.

3. Garcia-Guimaraes M, Bastante T, Antuna P et al. Spontaneous Coronary Artery Dissection: Mechanisms, Diagnosis and Management. Eur Cardiol 2020;15:1-8.
